# Effects of Different Light Intensities on the Growth and Photosynthetic Physiological Characteristics of *Cremastra appendiculata* (D. Don) Makino Seedlings

**DOI:** 10.3390/plants15030388

**Published:** 2026-01-27

**Authors:** Bingyan Liu, Siwen Wang, Jingjing Li, Jie Wang, Xinyue Hou, Yue Zhang, Liang Wang

**Affiliations:** 1 College of Landscape Architecture, Changchun University, Changchun 130022, China; lby0620@126.com (B.L.); 13630643305@163.com (S.W.); 18330815511@163.com (J.L.); 15829539117@163.com (J.W.); 19508689752@163.com (X.H.); 15585665610@163.com (Y.Z.); 2 Jilin Academy of Agricultural Sciences (Northeast Agricultural Research Center of China), Changchun 130033, China

**Keywords:** light intensity, seedling growth, leaf anatomical structure, photosynthetic performance, chlorophyll fluorescence

## Abstract

*Cremastra appendiculata* (D. Don) Makino, a rare orchid prized for its ornamental and medicinal value, exhibits high sensitivity to light conditions during the seedling stage. To identify optimal light intensity for promoting seedling growth and elucidate the underlying physiological mechanisms, this study exposed *C. appendiculata* seedlings to three light treatments: low light (LL, 80% shading, 300–350 µmol·m^−2^·s^−1^), medium light (ML, 60% shading, 600–650 µmol·m^−2^·s^−1^), and high light (HL, 30% shading, 900–1000 µmol·m^−2^·s^−1^). Growth and photosynthetic physiological parameters were measured to investigate the regulatory effects of light intensity. Results showed that under LL treatment, plant height, leaf area, and total biomass were significantly higher than those under HL treatment, increasing by 48%, 41%, and 50%, respectively. Leaf anatomical structure under LL displayed tightly arranged epidermal cells and intact mesophyll organization, consistent with typical shade-leaf characteristics. Chlorophyll content analysis revealed that chlorophyll a, chlorophyll b, and total chlorophyll under LL increased significantly by 75%, 35%, and 50%, respectively, compared to HL. Moreover, net photosynthetic rate peaked under LL, exceeding ML and HL by 28% and 17%, respectively. Chlorophyll fluorescence analysis further indicated that LL treatment optimized PSII performance, enhancing maximum photochemical efficiency, photosynthetic performance index, and electron transport rate per reaction center, while maintaining low thermal dissipation, indicating superior light capture and conversion efficiency. In summary, within the experimental gradient established in this study, the LL treatment represents the optimal light environment for the growth of *C. appendiculata* seedlings. By synergistically promoting plant morphological development, optimizing leaf structure, enhancing photosynthetic pigment content, and improving Photosystem II performance, this treatment facilitates efficient biomass accumulation. These findings provide a critical theoretical basis for the light environment management in both the conservation and artificial propagation of *C. appendiculata*.

## 1. Introduction

*Cremastra appendiculata* (D.Don) Makino is a rare perennial medicinal plant within the Orchidaceae family, possessing significant medicinal and ornamental value [1]. Its dried pseudobulbs, utilized for medicinal purposes, exhibit a range of pharmacological activities, including antitumor, anti-inflammatory, and antioxidant effects [2]. The seeds of *C. appendiculata* are minute and lack endosperm; under natural conditions, their germination depends on specific symbiotic fungi for nutrient supply, resulting in extremely low natural seed setting and germination rates [3]. Consequently, the efficient cultivation of *C. appendiculata* seedlings has become a critical limiting factor for industrial development. In intensive seedling production systems, light is a crucial environmental factor. It not only supplies energy for photosynthesis but also serves as a key signal regulating plant morphogenesis, physiological metabolism, and the accumulation of secondary metabolites [4]. Appropriate light intensity can promote plant photosynthesis, enhance light energy utilization efficiency, improve plant stress resistance, and thereby foster robust growth [5]. During the seedling stage, plant tissues are relatively tender, photosynthetic organs are not fully developed, and plants are more sensitive to light. Unsuitable light intensity can easily induce photoinhibition or lead to insufficient light energy utilization, subsequently restricting biomass accumulation and the robust growth of plants [6,7,8].

Light intensity not only affects the synthesis and distribution of photosynthetic pigments but also regulates photosynthetic physiological characteristics such as gas exchange parameters and chlorophyll fluorescence parameters, thereby exerting profound effects on biomass accumulation and morphogenesis [9]. Suitable light intensity can activate photosystem II (PSII), enhance photosynthetic electron transport efficiency, promote the synthesis of photosynthetic pigments and the development of thylakoid membranes, and improve light energy utilization efficiency [10]. However, when light intensity exceeds the regulatory range, photosynthetic machinery is at risk of photodamage, manifested as inactivation and disruption of PSII reaction centers, accumulation of reactive oxygen species, induction of photoinhibition, and consequent limitation of seedling biomass accumulation and morphogenesis [11]. The core of light intensity regulation lies in its influence on the photochemical performance and energy allocation strategies of leaves. Both excessively high and excessively low light intensities are detrimental to PSII activity, with overly strong light leading to photoinhibition [12]. Studies have shown that when light intensity exceeds 800 μmol·m^−2^·s^−1^, the PSII activity in *Panax notoginseng* (Burkill) F. H. Chen seedlings significantly decreases, with the Fv/Fm ratio potentially dropping below 0.6. Conversely, when light intensity falls below 100 μmol·m^−2^·s^−1^, the electron transport rate markedly slows, and photosynthetic efficiency declines [13,14]. Therefore, optimizing light intensity can improve the efficiency of light energy capture, transfer, and conversion in crops and medicinal plants, alleviate the pressure on photoprotective mechanisms, supply energy for carbon assimilation, and promote dry matter accumulation.

Currently, research on the effects of light on orchid growth predominantly focuses on mature plants or common ornamental varieties, whereas systematic studies on the photosynthetic physiological characteristics of seedlings from rare medicinal orchid species such as *C. appendiculata* remain limited. Unlike common orchid species, *C. appendiculata* seedlings exhibit a distinctive pseudobulb developmental pattern and a strong dependence on symbiosis, suggesting that their light-response strategy may be more inclined toward efficient utilization of low-light conditions to sustain early-stage survival and biomass accumulation. Consequently, this study was designed to not only identify the optimal light intensity for *C. appendiculata* seedlings but also to elucidate their coordinated adaptive mechanisms under low-light conditions from multiple dimensions, including leaf anatomy, photosynthetic performance, and photosystem function. The findings are expected to provide a theoretical basis for conservation-oriented seedling cultivation and light-environment management of this species.

## 2. Materials and Methods

### 2.1. Overview of the Test Area

The experiment was conducted at the Hongshi Forestry Bureau Entrepreneurial Incubation Demonstration Base (126°42′ E, 42°57′ N) in Hongshilazi Town, Huadian City, Jilin Province, China. The site experiences a temperate continental monsoon climate with four distinct seasons. The mean annual temperature is 3.9 °C, the average annual sunshine duration is 2379 h, and the mean annual precipitation is 748.1 mm. The soil at the experimental site is predominantly humus soil.

### 2.2. Experimental Design

**Figure 1:**
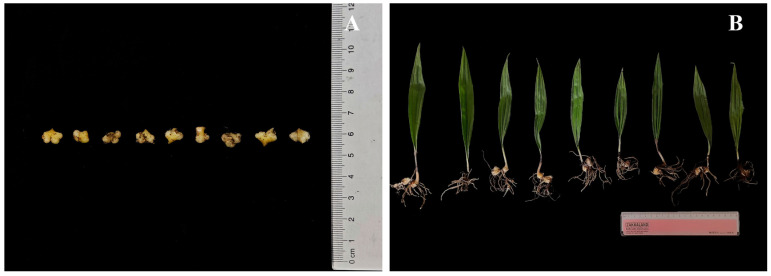
(**A**) *Cremastra appendiculata* (D. Don) Makino protocorm; (**B**) *C. appendiculata* seedlings.

The experiment began in early December 2023. Initially, plump and healthy capsules of *C. appendiculata* were selected, and the seed pods were opened. The seeds were then evenly mixed with a *C. appendiculata* inoculant, placed into inoculant bags, and incubated in a cultivation chamber at 25 °C [15]. Upon protocorm germination (Figure 1A) in early March 2024, they were evenly sown at a density of approximately 800 individuals per square meter onto artificially constructed square seedbeds with a side length of 2 m. Immediately after sowing, shading treatments were applied using shade nets of different densities, establishing three light intensity levels: low light (LL, 80% shading), medium light (ML, 60% shading), and high light (HL, 30% shading), with three replicates for each light treatment group. The photosynthetic photon flux density (PPFD) of each treatment was measured three times using a LI-190R quantum sensor (LI-COR Biosciences, Lincoln, NE, USA) at 10:00 AM on clear days during the initial experimental stage. The averaged values were as follows: 300–350 µmol·m^−2^·s^−1^ for the LL treatment, 600–650 µmol·m^−2^·s^−1^ for the ML treatment, and 900–1000 µmol·m^−2^·s^−1^ for the HL treatment. The shade nets were secured to frames positioned 2 m above the seedbeds to ensure uniform shading across all treatments. Consistent water and nutrient management were maintained throughout the growth period. In late August 2025, fifteen robust and uniformly growing *C. appendiculata* seedlings (Figure 1B) were randomly selected from each treatment for the measurement of growth parameters, leaf anatomical structure, and chlorophyll content. Gas exchange parameters and chlorophyll fluorescence characteristics were measured in situ. The entire experimental period, from the initiation of symbiotic germination to final sampling, lasted approximately 20 months.

### 2.3. Measurement Methods

#### 2.3.1. Observation of Leaf Tissue Anatomical Structure

Sampling for this experiment was conducted between 9:00 and 10:00 AM under clear weather conditions. Nine plants with uniform growth and no obvious pests or diseases were selected across the treatments. From the middle part of the leaves of these nine samples, avoiding the main vein, approximately 5 mm × 5 mm segments were excised using a scalpel. The segments were immediately fixed in glutaraldehyde solution (Sinopharm Chemical Reagent Co., Ltd., Shanghai, China), followed by dehydration through an ethanol gradient (Sinopharm Chemical Reagent Co., Ltd., Shanghai, China) and substitution with tertiary butanol (Sinopharm Chemical Reagent Co., Ltd., Shanghai, China) [16]. After complete sublimation drying of the samples and tertiary butanol in a desiccator, the specimens were sputter-coated with gold. Observations and imaging of the leaf epidermis and cross-sections were performed using a scanning electron microscope, JSM-6510 (JEOL Ltd., Tokyo, Japan). Imaging was conducted in secondary electron mode. To achieve optimal image quality for different sample surface heights, the working distance was adjusted within a range of 6–10 mm. Other parameters were consistently set as follows: accelerating voltage 15 kV, scan speed 6, and magnification 300×. Relevant indicators were subsequently measured using the image analysis software ImageJ 1.53t (National Institutes of Health, Bethesda, MD, USA).

The observed and quantified indicators primarily included stomatal status, leaf anatomical structures, and derived parameters. Stomatal status was directly recorded through observation; field of view area, upper epidermal thickness (UET), lower epidermal thickness (LET), leaf thickness (LT), palisade tissue thickness (PT), and spongy tissue thickness (ST) were obtained via direct measurement. Based on the measured data, stomatal density (SD), palisade-to-spongy tissue ratio (P/S), leaf tissue compactness (CTR), and leaf tissue sponginess (SR) were calculated using the following formulas:

Stomatal density = Number of stomata within the field of view/Area of the field of view

Palisade-to-spongy tissue ratio = Palisade tissue thickness/Spongy tissue thickness

Leaf tissue compactness = (Palisade tissue thickness/Leaf thickness) × 100%

Leaf tissue sponginess = (Spongy tissue thickness/Leaf thickness) × 100%

#### 2.3.2. Determination of Growth Indicators

For this experiment, a total of 27 leaf samples were collected, with nine leaves randomly selected from each treatment. Leaf area was measured by scanning the leaves using an AM-300 handheld leaf area meter (Zhejiang Top Cloud-Agri Technology Co., Ltd., Hangzhou, China). Plant height and stem base diameter of *C. appendiculata* were measured with a ruler and a vernier caliper, respectively. After the measurements, leaves, pseudobulbs, and roots were separated, excised, and placed into corresponding paper boxes. The samples were first fixed at 120 °C in an oven, then dried at 80 °C until constant weight was achieved. The biomass of each organ was weighed using an analytical balance, and the Seedling Quality Index (SQI) was calculated [17].

SQI = Total seedling biomass/(Height-to-root collar diameter ratio + Shoot-to-root biomass ratio)

#### 2.3.3. Determination of Chlorophyll Content

For this experiment, a total of nine healthy leaf samples were collected, with three leaves randomly selected from each treatment. The leaves were rinsed with distilled water to remove surface contaminants and gently blotted dry. Leaf segments were excised avoiding the midrib, and samples from the same treatment were pooled and finely chopped. Then, 0.2 g of the mixed sample was weighed and rapidly ground into a homogeneous paste under dim light. The homogenate was diluted to a final volume of 25 mL with 96% ethanol and stored in the dark for 24 h. Chlorophyll content was quantified using a UV-8000 A ultraviolet-visible spectrophotometer (Shanghai Mapada Instruments Co., Ltd., Shanghai, China) by measuring the absorbance at 649 nm and 665 nm in triplicate [18]. The calculation formulas used are as follows:

Chl a = (13.59 × A_665_ − 6.88 × A_649_) × V/1000 m

Chl b = (24.96 × A_649_ − 7.32 × A_665_) × V/1000 m

Chl (a + b) = Chl a + Chl b

#### 2.3.4. Determination of Photosynthetic Performance

Measurements were conducted between 9:00 and 11:00 AM on clear days. Net photosynthetic rate (Pn), stomatal conductance (Gs), intercellular CO_2_ concentration (Ci), and transpiration rate (Tr) of the middle section of *C. appendiculata* leaves were synchronously measured using a LI-6800 portable photosynthesis system (LI-COR Biosciences, Lincoln, NE, USA). The measurement environment was set with a CO_2_ concentration of 400 µmol·mol^−1^, a relative air humidity of 75%, and a leaf-chamber temperature of 25 °C. The built-in red-blue light source of the instrument was used to simulate the preset light environments, with photosynthetic photon flux density (PPFD) set to 300 µmol·m^−2^·s^−1^ for the LL treatment, 600 µmol·m^−2^·s^−1^ for the ML treatment, and 900 µmol·m^−2^·s^−1^ for the HL treatment. Data were recorded after photosynthetic parameters stabilized, with three leaves measured per treatment and the average value taken.

The chlorophyll fluorescence module integrated into this instrument was further employed to acquire chlorophyll fluorescence induction kinetics curves (OJIP curves) and associated parameters. Each shading treatment was fully dark-adapted for 20 min before measurement. The fluorescence measurement settings were as follows: continuous actinic light was turned off (Actinic Light = 0 µmol·m^−2^·s^−1^); high-intensity saturation pulse light (Saturation Pulse, SPS) at 8000 µmol·m^−2^·s^−1^ for 1.0 s was applied to induce the OJIP transient; measuring light was maintained at 0.5 µmol·m^−2^·s^−1^. All measurements were conducted under red light (625 nm). The OJIP curve comprises four standard characteristic points: O (initial fluorescence), J, I, and P (maximum fluorescence). Among these, the O and P points correspond to the fluorescence intensity at the start of the measurement (0 ms) and at approximately 1000 ms after saturation with actinic light, respectively. The J and I points were automatically identified by the instrument’s built-in software as the two main inflection points during the rapid-rise phase of fluorescence induction kinetics, with typical occurrence times around 2 ms and 30 ms, respectively. The OJIP curve was then normalized over the O–J and O–P phases, and the following key fluorescence parameters were derived based on the JIP-test analysis: maximum photochemical efficiency (Fv/Fm), total photosynthetic performance index (PItotal), performance index based on absorbed light energy (PIABS), as well as excitation energy allocation parameters per reaction center and per unit leaf area [19].

### 2.4. Statistical Analysis

The experimental data were organized and initially processed using Microsoft Excel 2020 (Microsoft Corporation, Redmond, WA, USA). Subsequently, statistical analyses were performed with IBM SPSS Statistics 22 (IBM Corporation, Armonk, NY, USA). Graphical visualizations were created using OriginPro 2021 (OriginLab Corporation, Northampton, MA, USA). For principal component analysis, all growth, structural, and photosynthetic physiological indicators were standardized using Z-score normalization to ensure that each variable contributed equally to the analysis.

## 3. Results Analysis

### 3.1. Effects of Different Light Intensities on the Leaf Anatomical Structure of C. appendiculata

**Figure 2:**
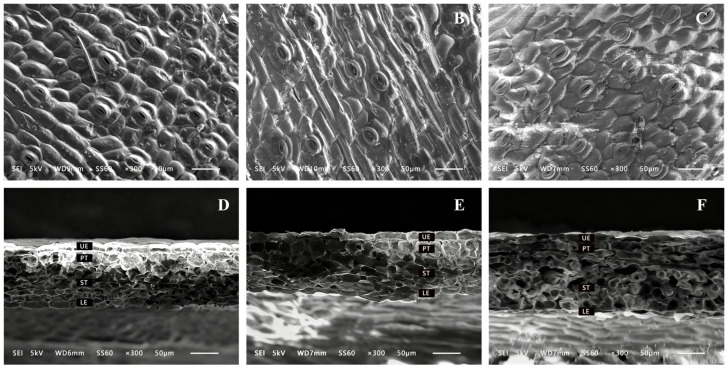
Microstructure of stomata and mesophyll in the lower epidermis of *C. appendiculata* leaves grown under different light intensities (×300). (**A**) Stomata in the lower epidermis under LL; (**B**) Stomata in the lower epidermis under ML; (**C**) Stomata in the lower epidermis under HL; (**D**) Mesophyll structure under LL; (**E**) Mesophyll structure under ML; (**F**) Mesophyll structure under HL. UE: Upper epidermis; PT: Palisade tissue; ST: Spongy tissue; LE: Lower epidermis.

Scanning electron microscopy images revealed the microstructural changes in the surface and cross-sections of *C. appendiculata* leaves (Figure 2). It can be clearly observed that the lower epidermal cells of *C. appendiculata* leaves exhibit a typical polygonal structure, predominantly quadrangular to hexagonal, with densely arranged adjacent cells. Stomata are composed of two kidney-shaped guard cells, aligned in coordination with surrounding epidermal cells. Additionally, well-defined palisade and spongy tissues are present. In the epidermal structure under the LL treatment, dense and regular scaly protrusions were observed, arranged in a compact and uniform pattern. The cross-sectional structure showed that the LL treatment resulted in clear and tightly organized tissue layers, with the upper epidermis, palisade tissue, spongy tissue, and lower epidermis all arranged in an orderly manner. In contrast, the ML and HL treatments exhibited disorganization in both upper and lower epidermal cells, along with blurred palisade tissue layers and expanded pores in the spongy tissue. These findings indicate that different light intensities significantly affect the microstructure of *C. appendiculata* leaves. As light intensity decreased, the leaf structure tended to exhibit characteristics of “shade leaves,” while the ML and HL treatments may have impacted physiological processes such as photosynthesis and gas exchange.

With decreasing light intensity, parameters including stomatal density, upper epidermal thickness, lower epidermal thickness, total leaf thickness, palisade tissue thickness, and spongy tissue thickness all showed a significant declining trend (Table 1). Compared with the HL treatment, the LL treatment exhibited reductions of 20.56%, 26.92%, 23.42%, 24.85%, 33.34%, and 33.73%, respectively. The palisade-to-spongy tissue ratio and mesophyll tissue compactness reached their highest values under the ML treatment and were significantly greater than those under the LL and HL treatments. No significant difference in mesophyll tissue looseness was observed between the ML and HL treatments, although both were higher than that under the LL treatment.

### 3.2. Effects of Different Light Intensities on the Growth of C. appendiculata Seedlings

**Figure 3:**
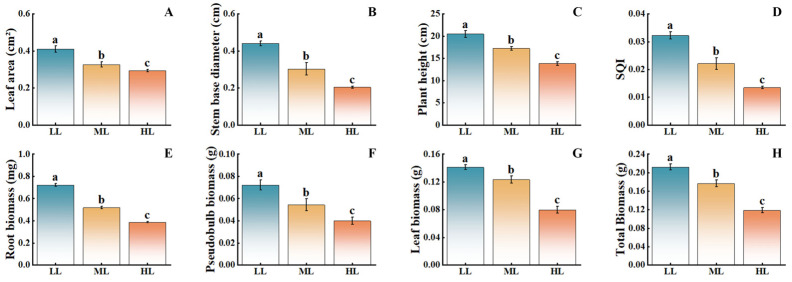
Effects of different light intensities on the growth of *C. appendiculata* seedlings. (**A**) Leaf area; (**B**) Stem base diameter; (**C**) Plant height; (**D**) Seedling quality index (SQI); (**E**) Root biomass; (**F**) Pseudobulb biomass; (**G**) Leaf biomass; (**H**) Total biomass. Different letters indicate statistical differences *p* < 0.05).

As light intensity decreased, the growth indicators of *C. appendiculata* seedlings showed a significant upward trend (Figure 3). Compared with the HL treatment, the LL treatment resulted in increases of 41.38% in leaf area, 120.00% in stem base diameter, 48.01% in plant height, 84.62% in root biomass, 75.00% in pseudobulb biomass, and 75.00% in leaf biomass, with the ML treatment exhibiting intermediate values. These findings indicate that low-light conditions are more conducive to the vegetative growth of this plant, while moderate and high light intensities inhibit its growth, with the inhibitory effect becoming more pronounced as light intensity increases.

### 3.3. Effects of Different Light Intensities on Photosynthetic Pigments and Gas Exchange Parameters of C. appendiculata Seedlings

**Figure 4:**
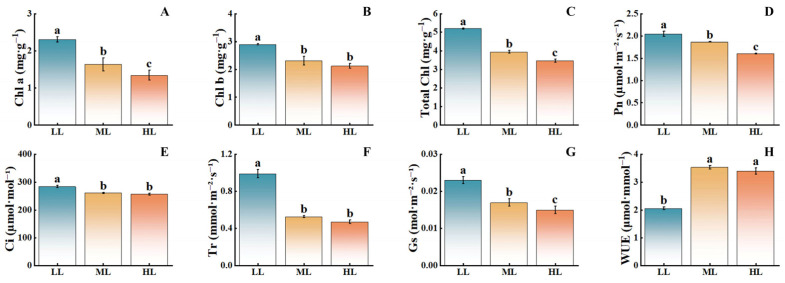
Effects of different light intensities on the photosynthetic performance of *C. appendiculata* seedlings. (**A**) Chlorophyll a; (**B**) Chlorophyll b; (**C**) Total Chlorophyll; (**D**) Net photosynthetic rate (Pn); (**E**) Intercellular carbon dioxide concentration (Ci); (**F**) Transpiration rate (Tr); (**G**) Stomatal conductance (Gs); (**H**) Water use efficiency (WUE). Different letters indicate statistical differences (*p* < 0.05).

With decreasing light intensity, the chlorophyll content in *C. appendiculata* seedlings significantly increased (Figure 4). Compared with the HL treatment, the LL treatment showed significant increases in chlorophyll a, chlorophyll b, and total chlorophyll by 74.63%, 34.86%, and 50.00%, respectively. Net photosynthetic rate, stomatal conductance, intercellular CO_2_ concentration, and transpiration rate also exhibited significant rises, increasing by 27.5%, 53.33%, 10.69%, and 110.02%, respectively, while water use efficiency significantly decreased, with the ML treatment displaying intermediate values. Notably, the trend of net photosynthetic rate closely aligned with that of stomatal conductance, suggesting that changes in gas exchange parameters may be primarily regulated by stomatal behavior. The positive correlation between net photosynthetic rate and chlorophyll content further revealed a coordinated variation between the functional output of the photosynthetic apparatus and its material foundation. These results indicate that low light intensity can significantly enhance chlorophyll content in *C. appendiculata* seedlings, thereby improving photosynthetic efficiency, while high light intensity exerts a pronounced inhibitory effect.

### 3.4. Effects of Different Light Intensities on Chlorophyll Fluorescence Parameters and Photochemical Performance of C. appendiculata Seedlings

**Figure 5:**
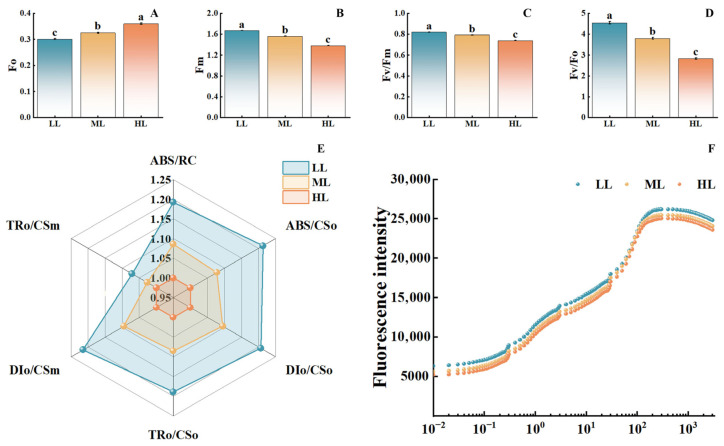
Effects of different light intensities on chlorophyll fluorescence parameters of *C. appendiculata* seedlings. (**A**) Initial fluorescence (Fo); (**B**) Maximum fluorescence (Fm); (**C**) Maximum photochemical efficiency of photosystem II (Fv/Fm); (**D**) Potential photochemical activity (Fv/Fo); (**E**) Energy allocation parameters of PSII reaction centers; (**F**) OJIP Curves. Different letters indicate statistical differences (*p* < 0.05).

As light intensity increased, the initial fluorescence significantly rose (Figure 5). The HL treatment showed an approximately 20% increase compared to the LL treatment. In contrast, maximum fluorescence, the maximum photochemical efficiency of PSII, and the potential photochemical activity all exhibited a significant declining trend. Compared to the LL treatment, the HL treatment demonstrated reductions of approximately 23%, 16%, and 35% in these parameters, respectively, indicating that high light intensity damages the structure and function of PSII. The radar chart of energy allocation parameters in the PSII reaction centers (Figure 5E) demonstrated that the photosynthetic performance parameters under the LL treatment covered the widest area, whereas those under the HL treatment covered the smallest. Furthermore, in the OJIP curves (Figure 5F), the fluorescence intensity of the LL treatment consistently exceeded that of the ML and HL treatments, with the HL treatment showing the smallest amplitude of curve rise. These findings further confirm that high light intensity inhibits the photosynthetic electron transport process. Overall, increased light intensity significantly impairs PSII function, reduces photochemical activity and photosynthetic electron transport efficiency, with the inhibitory effect being most pronounced under high light conditions.

### 3.5. Principal Component Analysis (PCA) and Correlation Analysis

**Figure 6:**
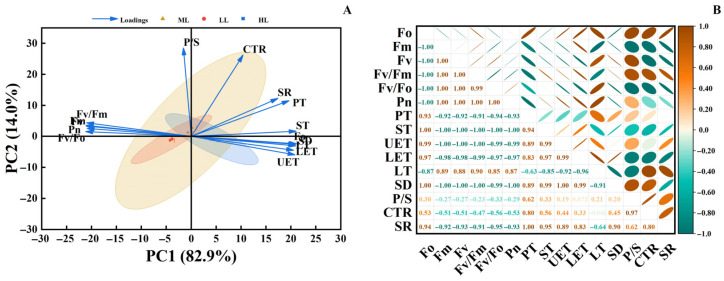
(**A**) Principal component analysis (PCA); (**B**) Correlation heatmap.

Principal component analysis (PCA) presented in Figure 6A shows that PC1 (82.9%) and PC2 (14.0%) together explain 96.9% of the total variance. Sample points from different light treatments exhibit distinct clustering. Photosynthetic indicators (e.g., Fv/Fm, Pn) show a negative correlation with PC1, while leaf structural indicators (e.g., P/S, PT) are positively correlated with PC1, reflecting a divergence between these two types of variables. The correlation heatmap in Figure 6B further indicates strong positive correlations among photosynthetic parameters and relatively strong positive correlations among leaf structural parameters. In contrast, most correlations between these two categories are negative, and correlations involving mesophyll structural parameters with other indicators are relatively weak. Collectively, these results demonstrate that light intensity serves as a key environmental factor driving the observed differences in photosynthetic traits and leaf structure in *C. appendiculata* seedlings, revealing a pronounced trade-off relationship between these two functional dimensions.

## 4. Discussion

### 4.1. Effects of Light Intensity on the Leaf Anatomical Structure of C. appendiculata Seedlings and Its Adaptive Strategies

As the core organ for plant–environment interaction, leaf anatomical structure directly reflects adaptation to light conditions [20]. In this study, leaves of *C. appendiculata* under LL treatment exhibited dense and regularly arranged scale-like epidermal protrusions, along with compact mesophyll tissue layers, consistent with the leaf structural features reported for *Dendrobium officinale* seedlings under low-light conditions. These morphological traits enhance light capture under dim environments by increasing epidermal surface roughness, while tightly organized palisade and spongy tissues minimize light scattering and energy loss within intercellular spaces [21,22]. In contrast, under ML and HL treatments, disorganization of epidermal cells and collapsed spongy tissue pores were observed, aligning with findings on leaf cell ultrastructural damage in *Anoectochilus roxburghii* seedlings under high light intensity [23]. This suggests that under low light conditions, *C. appendiculata* optimizes leaf structure to improve light capture and utilization efficiency, while under high light conditions, leaf structure tends to deteriorate due to oxidative stress, reflecting a trade-off between resource utilization and self-protection in plants.

From the perspective of quantitative structural indicators, as light intensity increased, the stomatal density, epidermal thickness, and mesophyll tissue thickness of *C. appendiculata* leaves significantly increased. This represents a structural compensatory strategy adopted by plants to cope with high-light stress. Thickened epidermis reduces direct light radiation into mesophyll cells, while increased stomatal density attempts to alleviate constraints on photosynthetic carbon assimilation by enhancing gas exchange rates [20]. However, in the present study, the palisade-to-spongy tissue ratio decreased under the HL treatment. This may be attributed to the immature development of photosynthetic organs in *C. appendiculata* seedlings, where the expansion of palisade tissue cells was restricted under high light, while the spongy tissue became excessively loose, ultimately disrupting the structural balance of the mesophyll. This alteration in leaf structure likely represents a form of self-protective adaptation, in which thicker epidermal layers and looser internal tissue help reduce direct penetration of intense light and modulate gas exchange, thereby mitigating high-light damage. The decline in photosynthetic performance can be explained by the inability of the photosynthetic apparatus to supply sufficient CO_2_ to process the excess reductants generated under such high light intensity. Therefore, the observed structural modifications in leaves may serve to regulate this physiological imbalance. In natural environments, plants often close their stomata during midday under intense light to limit water loss and avoid photodamage, while efficient photosynthesis is primarily confined to periods of suitable light conditions in the morning and evening, thereby explaining the compromised photosynthetic performance observed under HL treatment despite increased leaf thickness.

### 4.2. Effects of Light Intensity on Chlorophyll Content in C. appendiculata and Its Physiological Implications

Chlorophyll serves as the material basis for light energy capture in plants, with its content and composition directly determining photosynthetic efficiency [24]. In the present study, the contents of chlorophyll a, chlorophyll b, and total chlorophyll under the LL treatment were significantly higher than those under the ML and HL treatments. This aligns with the pigment accumulation pattern observed in seedlings of saprophytic orchids, such as *Gastrodia elata*, under low light conditions [25]. In low-light environments, plants enhance their light-capturing capacity by upregulating chlorophyll synthesis and increasing pigment content [26]. These findings suggest that *C. appendiculata* seedlings may adjust chlorophyll content and composition under high-light stress to balance the requirements of light capture and photoprotection.

### 4.3. Regulatory Mechanisms of Light Intensity on Gas Exchange Parameters in C. appendiculata Seedlings

Changes in gas exchange parameters further reveal the regulatory effects of light intensity. The synchronous increase in net photosynthetic rate and stomatal conductance under the LL treatment indicates that under low-light conditions, *C. appendiculata* seedlings enhance CO_2_ supply by opening stomata while utilizing high chlorophyll content to improve light capture, forming a synergistic mechanism for photosynthetic promotion [27]. In contrast, under the HL treatment, the decline in net photosynthetic rate is accompanied by a decrease in intercellular CO_2_ concentration, suggesting that the photosynthetic inhibition is primarily due to stomatal limitation at this stage [28], a finding consistent with results observed in *Syringa oblata* under high light, where stomatal closure leads to reduced photosynthesis [29]. Notably, the transient increase in water use efficiency under the ML treatment reflects a physiological adaptation to moderate stress, where plants reduce water loss by decreasing transpiration rates. However, this adaptation does not persist under the HL treatment, likely due to excessive stomatal closure under high light, which limits CO_2_ supply and ultimately causes water use efficiency to revert to levels similar to those under the LL treatment. This pattern closely aligns with the water physiological responses observed in Bletilla striata seedlings [30].

### 4.4. Analysis of Chlorophyll Fluorescence Characteristics, PSII Function, and Photosynthesis-Structure Trade-Offs in C. appendiculata Seedlings

Chlorophyll fluorescence parameters, serving as probes for the function of the photosynthetic system, more directly reflect the effects of light intensity on PSII [31]. In this study, the increase in Fo and the decrease in Fv/Fm and Fv/Fo under the HL treatment are typical indicators of PSII reaction center inactivation [32], which aligns with findings on PSII damage in Dendrobium under high light [33]. Conversely, the synergy between low thermal dissipation and high electron transport rates under the LL treatment indicates that under low-light conditions, *C. appendiculata* seedlings allocate more light energy to photochemical reactions, reducing energy loss due to non-photochemical quenching. This represents a core physiological mechanism for its adaptation to low-light environments.

Principal component and correlation analyses collectively reveal a distinct “photosynthesis-structure trade-off” strategy in *C. appendiculata* seedlings under varying light conditions. Under low-light conditions, plants prioritize the optimization of photosynthetic physiological functions—such as pigment synthesis and PSII activity—to maximize light-use efficiency, while maintaining basic leaf structural integrity sufficient to meet functional demands. Conversely, under high-light conditions, plants attempt to mitigate photodamage by thickening leaf structures (e.g., epidermis, palisade tissue). However, such structural compensation requires substantial consumption of photosynthetic assimilates, and the direct photodamage to the photosynthetic apparatus cannot be fully offset by structural adjustments alone. This ultimately results in a trade-off outcome characterized by “structural thickening accompanied by diminished physiological activity” [34]. Compared with studies on the light response of *Cypripedium macranthos* Sw. seedlings [35], *C. appendiculata* exhibits a lower trade-off threshold and higher efficiency in low-light utilization, which may be associated with its native understory habitat characterized by stable low-light conditions and its survival strategy favoring rapid early-stage biomass accumulation. This finding not only elucidates the unique light-resource allocation strategy in *C. appendiculata* but also provides differentiated light-management guidance for its artificial cultivation and conservation, distinct from that for common orchid species.

In summary, the adaptation of *C. appendiculata* seedlings to low-light environments results from the synergistic effects of “structural maintenance and physiological optimization.” An 80% shading treatment ensured leaf structural integrity, promoted chlorophyll synthesis, and optimized PSII function, thereby achieving efficient light capture and conversion. These findings not only provide technical guidance for the artificial cultivation of *C. appendiculata* seedlings but also offer a reference framework for light environment regulation in other rare medicinal orchid species.

## 5. Conclusions

This study investigated the effects of different light intensities on leaf anatomical structure, growth characteristics, and photosynthetic physiology of *C. appendiculata* seedlings, aiming to identify optimal light conditions and elucidate their response mechanisms to light intensity. The main conclusions of this study are as follows: Low light represents the optimal light environment for *C. appendiculata* seedlings. Under this treatment, the seedling leaves maintained a scale-like epidermal structure and compact mesophyll tissue organization, ensuring efficient photosynthesis and gas exchange. Growth parameters, including plant height, leaf area, and total biomass, were all significantly higher under LL treatment compared to ML and HL treatments. This indicates that LL provides the most favorable conditions for its vegetative growth. Light intensity significantly regulated photosynthetic performance. Under the LL treatment, chlorophyll content significantly increased, and gas exchange parameters peaked. Chlorophyll fluorescence parameters and related data confirmed that LL treatment maximized PSII photochemical activity and electron transport efficiency, achieving efficient light capture and conversion, whereas HL treatment inhibited photosynthetic function.

In summary, this study systematically reveals the multidimensional adaptive advantages of *C. appendiculata* seedlings in low-light environments: through the synergistic optimization of photosynthetic performance and the maintenance of leaf structural integrity, efficient biomass accumulation is achieved under low light intensity. Therefore, within the light-intensity gradient established in this experiment, the low-light treatment can serve as a relatively optimal management strategy for promoting the growth of *C. appendiculata* seedlings under the current experimental conditions. Furthermore, given the positive growth trend observed in the study, it is necessary to incorporate even lower light-intensity gradients in future research to more precisely determine its light saturation point and identify the optimal light range for maximizing growth potential.

## Figures and Tables

**Table 1:** The effects of different light intensities on the leaves of *C. appendiculata* seedlings.

Item	LL	ML	HL
SD/mm^−2^	95.33 ± 2.51 c	105.00 ± 3.00 b	120.00 ± 1.00 a
UET/μm	14.47 ± 0.31 c	16.17 ± 0.25 b	19.80 ± 0.46 a
LET/μm	12.20 ± 0.36 c	13.60 ± 0.50 b	15.93 ± 0.42 a
LT/μm	113.47 ± 0.25 c	129.37 ± 2.72 b	151.00 ± 3.08 a
PT/μm	20.13 ± 0.55 c	28.50 ± 0.46 b	30.20 ± 0.26 a
ST/μm	53.63 ± 1.80 c	67.93 ± 2.53 b	80.93 ± 3.97 a
P/S	0.38 ± 0.01 b	0.42 ± 0.01 a	0.37 ± 0.02 b
CTR/%	0.18 ± 0.01 c	0.22 ± 0.01 a	0.20 ± 0.01 b
SR/%	0.47 ± 0.02 b	0.52 ± 0.03 a	0.53 ± 0.02 a

Note: Stomatal density (SD); Upper epidermis thickness (UET); Lower epidermis thickness (LET); Leaf thickness (LT); Palisade tissue thickness (PT); Spongy tissue thickness (ST); Palisade to spongy ratio (P/S); Leaf tissue compactness (CTR); Leaf tissue sponginess (SR). All data are expressed as mean ± standard deviation and different letters indicate significant differences between treatments (*p* < 0.05).

## Data Availability

The original contributions presented in this study are included in the article. Further inquiries can be directed to the corresponding author.
